# Behavioral and molecular processing of visceral pain in the brain of mice: impact of colitis and psychological stress

**DOI:** 10.3389/fnbeh.2015.00177

**Published:** 2015-07-10

**Authors:** Piyush Jain, Ahmed M. Hassan, Chintan N. Koyani, Raphaela Mayerhofer, Florian Reichmann, Aitak Farzi, Rufina Schuligoi, Ernst Malle, Peter Holzer

**Affiliations:** ^1^Research Unit of Translational Neurogastroenterology, Institute of Experimental and Clinical Pharmacology, Medical University of GrazGraz, Austria; ^2^Institute of Molecular Biology and Biochemistry, Medical University of GrazGraz, Austria

**Keywords:** cellular signaling, central nervous system, cerebral pain processing, colonic inflammation, emotional pain responses, intestinal nociception, psychological stress, somatic hypersensitivity

## Abstract

Gastrointestinal disorders with abdominal pain are associated with central sensitization and psychopathologies that are often exacerbated by stress. Here we investigated the impact of colitis induced by dextran sulfate sodium (DSS) and repeated water avoidance stress (WAS) on spontaneous and nociception-related behavior and molecular signaling in the mouse brain. DSS increased the mechanical pain sensitivity of the abdominal skin while both WAS and DSS enhanced the mechanical and thermal pain sensitivity of the plantar skin. These manifestations of central sensitization were associated with augmented c-Fos expression in spinal cord, thalamus, hypothalamus, amygdala and prefrontal cortex. While WAS stimulated phosphorylation of mitogen-activated protein kinase (MAPK) p42/44, DSS activated another signaling pathway, both of which converged on c-Fos. The DSS- and WAS-induced hyperalgesia in the abdominal and plantar skin and c-Fos expression in the brain disappeared when the mice were subjected to WAS+DSS treatment. Intrarectal allyl isothiocyanate (AITC) evoked aversive behavior (freezing, reduction of locomotion and exploration) in association with p42/44 MAPK and c-Fos activation in spinal cord and brain. These effects were inhibited by morphine, which attests to their relationship with nociception. DSS and WAS exerted opposite effects on AITC-evoked p42/44 MAPK and c-Fos activation, which indicates that these transduction pathways subserve different aspects of visceral pain processing in the brain. In summary, behavioral perturbations caused by colitis and psychological stress are associated with distinct alterations in cerebral signaling. These findings provide novel perspectives on central sensitization and the sensory and emotional processing of visceral pain stimuli in the brain.

## Introduction

Visceral pain is a symptom of many gastrointestinal disorders including inflammatory bowel disease and irritable bowel syndrome (Schirbel et al., 2010; Halpin and Ford, 2012). Functional gastrointestinal disorders with abdominal pain are frequently associated with central sensitization (Moshiree et al., 2006; Stabell et al., 2013) and psychopathologies that often are triggered or exacerbated by stress (Levy et al., 2006). These circumstances emphasize that visceral pain processing need be studied at the cerebral level, if efficacious therapeutics are to be developed (Mayer et al., 2008; Holschneider et al., 2011). However, the interaction between external stressors (e.g., psychological stress) and internal stressors (e.g., gastrointestinal inflammation and nociception) in the sensory and emotional dimensions of visceral pain is insufficiently understood.

The overall goal of the current study was to investigate the impact of experimental colitis and psychological stress on spontaneous and nociception-related behavior as well as molecular signaling in the mouse brain. Repeated water avoidance stress (WAS) was used as psychological stressor, since both acute and repeated WAS have been found to affect pain sensitivity in rodents (Bradesi et al., 2005; Schwetz et al., 2005). To study the interaction of WAS with an internal gastrointestinal stressor, we employed colitis induced by dextran sulfate sodium (DSS) (Dothel et al., 2013) which reproduces several pathophysiological features of inflammatory bowel disease, particularly ulcerative colitis (Sainathan et al., 2008; Perse and Cerar, 2012).

The first aim was to examine the interaction of DSS-induced colitis and WAS in mice with respect to inflammation, disease severity and behavior (locomotion, exploration, ingestion, motivation, and self-care). The second aim was to investigate whether DSS-induced colitis, WAS and combined WAS+DSS treatment are associated with a change in the mechanical and thermal pain sensitivity of the abdominal and plantar skin. Intestinal hyperalgesia is related to mechanical hypersensitivity of the abdominal skin, and this phenomenon of referred pain can be used as a measure of intestinal pain (Laird et al., 2001; Eijkelkamp et al., 2007). We further explored whether the behavioral alterations evoked by DSS and/or WAS are reflected by activation of p42/44 mitogen-activated protein kinase (MAPK) and c-Fos in the central nervous system (CNS). Both stress and gastrointestinal pain are known to cause cerebral p42/44 MAPK phosphorylation and expression of c-Fos, effects that have been linked to neuronal excitation, plasticity, and hypersensitivity (Michl et al., 2001; Obata and Noguchi, 2004; Obata et al., 2004; Eijkelkamp et al., 2007; Ji et al., 2009; Cargnello and Roux, 2011).

The third aim was to probe whether the behavioral and molecular CNS responses to acute colonic pain are affected by DSS-induced colitis and/or WAS. Colonic pain was provoked by intrarectal allyl isothiocyanate (AITC), which stimulates transient receptor potential ankyrin-1 and transient receptor potential vanilloid-1 channels on nociceptive nerve fibers (Everaerts et al., 2011; Holzer, 2011). AITC has been reported to evoke visceral pain behavior as well as p42/44 MAPK and c-Fos activation in the spinal cord (Laird et al., 2001; Mitrovic et al., 2010), but its effect on cerebral pain processing under conditions of colitis and psychological stress has not yet been systematically explored. We therefore examined whether intrarectal AITC causes nociception-related behavior (freezing, reduction of locomotion and exploration) in parallel with activation of p42/44 MAPK and c-Fos in the spinal cord and brain. The fourth aim was to address the question whether the AITC-evoked behavioral and molecular CNS responses are alleviated by an analgesic drug such as morphine, thus confirming the nociceptive nature of these effects (Mitrovic et al., 2010; Schwartz et al., 2013).

## Materials and methods

### Experimental animals

Male C57BL/6N mice (8 weeks old, 20–24 g) were obtained from Charles River (Sulzfeld, Germany) and habituated to the laboratory environment for at least 2 weeks. The animals were housed either one (*study* 2, see below) or two (*study* 1, 3, 4, 5, and 6, see below) per cage under controlled conditions of temperature (set point 21°C), air humidity (set point 50%) and a 12 h light/dark cycle (lights on at 6:00 a.m., lights off at 6:00 p.m.). Standard laboratory chow (altromin 1324 FORTI, Altromin, Lage, Germany) was provided *ad libitum* throughout the studies. All experiments were approved by an ethical committee at the Federal Ministry of Science and Research of the Republic of Austria (BMWF-66.010/0118-II/3b/2011 and BMWFW-66.010/0054-WF/II/3b/2014) and conducted according to the Directives 86/609/EEC and 2010/63/EU of the European Communities Council. The experiments were designed in such a way that both the number of animals used and their suffering was minimized.

### Study design

Six studies (*study* 1–6, Table 1) were carried out. In each study except *study* 6, mice were randomly allocated to four treatment groups: group I (control; no treatment), group II (WAS, subjected to intermittent WAS for 7 days), group III (DSS, treated with DSS for 7 days), and group IV (WAS+DSS, subjected to intermittent WAS and treated with DSS for 7 days). Group II animals were challenged with intermittent WAS by placing them 1 h/day (7 days) on a small platform (6×3 × 3 cm; length × width × height) in the center of a water-filled tank (50×32 × 30 cm; length × width × height) (Melgar et al., 2008). The water level in the tank was kept at 0.5 to 1 cm below the platform. Group III animals were treated with 2% (w/v) DSS (molecular weight 36,000–50,000; MP Biomedicals, Illkirch, France) in the drinking water for 7 days. Group IV animals underwent both the WAS challenge and DSS treatment for 7 days. The body weight of the animals was measured on day 1 before the start of any treatment and on day 8.

**Table 1:** **Experimental groups and study plan**.

	**Days 1–7**	**Day 8**	**Day 9**
*Study 1* (40 mice)	Control, WAS, DSS, WAS+DSS	Western blot analysis	
*Study 2* (28 mice)	Control, WAS, DSS, WAS+DSS	Recording of locomotion, exploration, and ingestion	
*Study 3* (32 mice)	Control, WAS, DSS, WAS+DSS	Splash test	
*Study 4* (46 mice)	Control, WAS, DSS, WAS+DSS	von Frey test	Plantar test
*Study 5* (80 mice)	Control, WAS, DSS, WAS+DSS	Intrarectal AITC instillation followed by recording of visceral pain behavior and Western blot analysis	
*Study 6* (32 mice)	Control	Intrarectal AITC instillation in the absence or presence of morphine followed by recording of visceral pain behavior and Western blot analysis	

After completion of the 7-day treatment period, the animals were randomly assigned to one of the following studies (Table 1). In *study* 1, the animals were euthanized by intraperitoneal (i.p.) injection of pentobarbital (150 mg/kg) on day 8; then spinal cords and brains were isolated, homogenized and subjected to Western blot analysis. The expression of p42/44 and phosphorylated p42/44 (pp42/44) MAPK and c-Fos was evaluated in the lumbosacral spinal cord and brain.

*Study* 2 and 3 were carried out to examine behavioral changes in response to the treatment regimens on day 8. In *study* 2, short-term activity (locomotion, exploration, and ingestion) for a period of 60 min was measured with the LabMaster system (TSE Systems, Bad Homburg, Germany). In *study* 3, the motivational and self-care behavior of animals was estimated with the splash test.

*Study* 4 was designed to assess somatic pain sensitivity of the abdominal and plantar region. On day 8, the von Frey hair test for mechanical pain sensitivity and on day 9 the plantar test for thermal pain sensitivity were performed.

*Study* 5 was carried out to examine the effect of intrarectal administration of AITC (Sigma-Aldrich) on visceral pain behavior and protein expression in the CNS of all four treatment groups (group I–IV). The animals received an intrarectal instillation (0.05 ml) of either AITC (2% v/v) in peanut oil (Sigma-Aldrich) or peanut oil (vehicle control for AITC). Immediately after AITC instillation, visceral pain behavior was recorded for 15 min. One hour post-AITC instillation, the mice were sacrificed and tissues collected.

*Study* 6 was carried out to examine the effect of intrarectal administration of AITC (Sigma-Aldrich, Vienna, Austria), alone or in combination with morphine (Gerot-Lannach, Lannach, Austria) premedication, on visceral pain behavior and protein expression in the CNS of control mice (group I). The animals received an intrarectal instillation (0.05 ml) of either AITC (2% v/v) in peanut oil (Sigma-Aldrich) or peanut oil (vehicle control for AITC). One hour prior to AITC treatment, the mice were treated with a subcutaneous injection (2 ml/kg) of either saline (0.9% NaCl in water, w/v) or morphine (10 mg/kg) in saline. Immediately after AITC instillation, visceral pain behavior was recorded for 15 min. One hour post-AITC instillation, the mice were sacrificed and tissues collected.

At the end of each study, the animals were euthanized by pentobarbital (150 mg/kg i.p.), and the severity of colitis was assessed by recording of a disease activity score. In addition, colon length (without stretching), weight (after cleaning), and colonic myeloperoxidase (MPO) mass were estimated. At the end of *study* 1, 5, and 6, the spinal cords and brains were isolated, homogenized, and subjected to Western blot analysis.

### Experimental protocols

#### Disease activity score

Following sacrifice, a disease activity score based on fur appearance and stool consistency was recorded. Normal and abnormal fur appearance scored 0 and 1, respectively. Normal, soft but formed and loose stool scored 0, 1, and 2, respectively, and the absence of bleeding or the presence of blood traces or gross bleeding in the perianal region scored 0, 1, and 2, respectively. The absence or presence of blood in the stool was evaluated with the Hemdetect® test (DIPROmed, Weigelsdorf, Austria) and scored as 0 or 1. The sum of all scores in each category yielded scores of 0 to 6 (Reichmann et al., 2013).

#### Colonic myeloperoxidase content

Myeloperoxidase mass in the distal colon was quantitated with a commercially available ELISA kit (Hycult Biotechnology, Uden, The Netherlands). Tissue samples were prepared according to the manufacturer's recommendations. Briefly, tissues were weighed and homogenized in lysis buffer (200 mM NaCl, 5 mM ethylenediaminetetraacetic acid, 10 mM trishydroxy methylaminomethane, 10% (v/v) glycerine, 1 mM phenylmethylsulphonyl fluoride (PMSF, Sigma-Aldrich P7626), 1 mg/ml leupeptide, and 28 mg/ml aprotinin, pH 7.4) at a tissue:lysis buffer ratio of 1 mg:0.02 ml. Tissue debris was pelleted by centrifugation (two runs at 6000 × g and 4°C for 15 min). Supernatants were used for measurement of MPO as marker for neutrophil infiltration. All standard and sample values were subtracted with blank control values, and MPO concentrations were quantitated using a standard curve. The sensitivity of the assay was 1 ng/ml at an intra- and inter-assay variation of around 10% (Reichmann et al., 2013).

#### Locomotor and ingestive activity

The mice were habituated to the water bottles used in the LabMaster system for 7 days before starting the 7-day treatment with WAS, DSS or WAS+DSS. On day 8, the activity (locomotion, exploration, and ingestion) of the animals was recorded with the LabMaster system. For this purpose, the mice were placed singly and without habituation in test cages (42×26.5×15 cm, length × width × height) which were connected to the LabMaster system for the recording of locomotion, exploration (rearing), feeding and drinking for 60 min without interruption (*study* 2). The LabMaster system was composed of six recording units consisting of six moveable cages surrounded by two horizontal infra-red frames and a cage lid with two weight transducers (Painsipp et al., 2013). Two frames positioned above each other allowed to record ambulatory (locomotion) and vertical (rearing, exploration) movements. The two weight transducers were used to record food and water intake. The recordings of ingestion were normalized to body weight.

#### Splash test

The splash test was used to assess grooming behavior (Taksande et al., 2013). On day 8, a 10% (w/v) sucrose solution (0.1 to 0.2 ml) in water was sprayed on the dorsal surface of the mice. Subsequently, the mice were placed in small plexiglass cages with tiny bedding (*study* 3). The cages were connected to the LabMaster system to record locomotion and rearing. Simultaneously, the behavior of the animals during the first 5 min was recorded with a video camera to evaluate grooming behavior. The video recordings were analyzed by a trained blinded observer with the VideoMot2 (TSE Systems) event monitoring module. The time taken to start grooming (either nose/face or head/body grooming) was measured and considered as an index of self-care while grooming frequency was used to assess motivational behavior (Taksande et al., 2013). A decrease in grooming frequency and an increase in grooming latency is indicative of low motivational and reduced self-care behavior (Taksande et al., 2013), respectively.

#### von Frey hair test

On day 8 (*study* 4), the mechanical pain sensitivity of the abdominal and plantar region was evaluated with von Frey filaments (Bioseb, Vitrolles, France) by a modification of the method of Laird et al. (2001). The mice were habituated in small compartments on a perforated grid (Dynamic Plantar Aesthesiometer, Ugo Basile, Comerio, Italy) for 1 h before the test. Subsequently von Frey filaments were applied to the abdomen (between diaphragm and genitals) and the plantar side of the right and the left hind paw. The test was performed by a trained blinded observer. The individual filaments were tested in an ascending order covering 0.008, 0.02, 0.04, 0.07, 0.16, 0.4, and 0.6 g forces. Each force was applied 10 times to the abdominal surface, and 5 times to the right and the left hind paw. The maximal duration of each force application was 2 s, and the inter-stimulus interval was 2–3 min. Following each challenge, the withdrawal response was quantified either as 1 (withdrawal of abdominal wall or paw, licking or retraction of animal) or 0 (no response). All counts in response to an individual filament were averaged. Withdrawal responses to low forces reflect high mechanical pain sensitivity.

#### Plantar test

On day 9 (*study* 4), the thermal pain sensitivity of the plantar region was evaluated with the Hargreaves (plantar) test using the Ugo Basile Thermal Paw Stimulator (Allen and Yaksh, 2004). Before the plantar test, the mice were habituated in the small compartments of the Ugo Basile Dynamic Plantar Aesthesiometer for 1 h before the test. Subsequently, the plantar side of the right and the left hind paw was exposed to an infrared source through a glass plate (cut-off time: 15 s). The test was performed by a trained blinded observer. The withdrawal response was assessed at three infrared intensities (1, 2, and 3; low to high intensity). The time taken by the mice to move their paw from the infrared light beam (withdrawal latency) was recorded. Each hind paw was subjected to 3 trials at an inter-stimulus interval of 5 min, and the average latency was calculated. A short latency of the withdrawal response reflects high thermal pain sensitivity.

#### Visceral pain behavior

Two dimensions of visceral pain behavior were simultaneously recorded after intrarectal administration of peanut oil or AITC (*study* 5 and 6). For this purpose, mice were placed in small plexiglass cages with tiny bedding. *Dimension* 1 was assessed by a modification of the method of Laird et al. (2001). In this test, the freezing behavior (typically associated with the AITC instillation) of the animals was recorded for 15 min with a video camera, and evaluated with the VideoMot2 (TSE Systems) event monitoring module by a blinded observer. *Dimension* 2 of the visceral pain behavior was a reduction of locomotion and rearing (Schwartz et al., 2013), which was recorded with the LabMaster system (TSE Systems) for 5 min. A decrease in locomotion and rearing is thought to reflect nociception-related behavior (Schwartz et al., 2013). Since the mice were not habituated to the test cages it cannot be ruled out that a component of novelty-induced behavior contributed to the recordings.

#### Tissue extraction and microdissection

After euthanization of the animals, the vertebral column was dissected from head to tail and cleaned. A 5 ml syringe filled with phosphate-buffered saline (PBS, pH 7.4) was used to flush the spinal cord from the caudal end of the vertebral column. The whole spinal cord was excised and the lumbosacral region was identified (Galan et al., 2003). Following dissection, the spinal cord was collected in homogenization tubes (Peqlab micro packaging vials, 2 ml, Peqlab Biotechnologie, Erlangen, Germany) containing 1.4 mm zirconium oxide beads (Peqlab Precellys beads), frozen in liquid nitrogen and stored at −70°C.

Brains were excised, placed on dry ice and stored at −70°C. Before microdissection, the brains were placed in a Microm HM 560 cryostat (Microm, Walldorf, Germany) at −20°C for 30 min. For microdissection, the brains were placed on a cold plate at −20°C (Weinkauf Medizintechnik, Forchheim, Germany), and the forebrains were cut into 8–10 slices. Subsequently the prefrontal cortex, hypothalamus, thalamus and amygdala were microdissected under a stereomicroscope and collected in homogenization tubes, placed on dry ice and stored at −70°C (Brunner et al., 2014).

#### Western blot analysis

The spinal cord and microdissected brain regions were homogenized in lysis buffer (50 mM Tris-HCl pH 8, 150 mM NaCl, 1% (v/v) Triton X-100, 0.5% (v/v) sodium deoxycholate, and 10 mM PMSF) and a Peqlab Precellys 24 homogenizer (two runs at 6500 rpm for 20 s with a 5 min refractory period). The tissue homogenates were centrifuged (10,000 rpm, 4°C, 10 min) to pellet debris. The protein content was measured with the BCA protein assay kit (Pierce Biotechnology, Rockford, IL, USA). Total protein lysates (50 μg) were mixed with 8 μl of 4xNuPAGE LDS sample buffer and 2 μl of NuPAGE sample reducing agent and heated for 10 min at 70°C. Protein samples were subjected to electrophoresis on 4–12% gradient SDS-PAGE gels. After transfer to nitrocellulose membranes (45 μm; Invitrogen, Lofer, Austria), proteins were visualized with Ponceau's solution (Sigma-Aldrich). Then the membranes were blocked with non-fat milk [5% (w/v) Tris-buffered saline containing Tween 20] (25°C, 2 h) (Rauh et al., 2008) and incubated overnight at 4°C with the following primary antibodies diluted in bovine serum albumin (5% (w/v) in Tris-buffered saline containing Tween 20): (i) anti-pp42/44 MAPK (1:1000, Cell Signaling-9106) and (ii) anti-c-Fos (1:1000, Cell Signaling-2250). After being washed, the membranes were incubated with horseradish peroxidase-conjugated goat anti-rabbit IgG (1:200,000, Biomol-6293, Hamburg, Germany) or goat anti-mouse IgG (1:100,000, Biomol-8101102). Immunoreactive bands were visualized with Super Signal West Pico Chemiluminescent substrate (Thermo Scientific, Vienna, Austria) and developed with the Bio-Rad ChemiDoc MP Imaging System (Bio-Rad, Vienna, Austria) (Kitz et al., 2011). For normalization, membranes were stripped (58.4 g/l NaCl, 7.5 g/l glycine, pH 2.15) and incubated with anti-p42/44 MAPK (1:2000, Santa Cruz SC-94, Heidelberg, Germany) or anti-glyceraldehyde 3-phosphate dehydrogenase (GAPDH) antibodies (1:1000, Santa Cruz SC-25778). Densitometric evaluations of immunoreactive bands were performed with the Image Lab software volume module.

### Statistics

The IBM SPSS 20 and SigmaPlot 12.5 packages were used to analyze and plot the results. Before applying any statistics, the data were checked for normal distribution with the Shapiro-Wilk test. The results of all experiments were analyzed by considering DSS treatment as one factor and WAS as the other factor. In experiments involving more than two treatment groups, two-way ANOVA or repeated measures ANOVA were used for statistical analysis, as appropriate. A DSS main factor effect was referred to by the term “DSS effect” (when combined data from the DSS and WAS+DSS groups were compared with combined data from the control and WAS groups). Analogously, a WAS main factor effect was denoted by “WAS effect” (when combined data from the WAS and WAS+DSS groups were compared with combined data from the control and DSS groups). Interactions between the two factors (two-way ANOVA) or three factors (repeated measures ANOVA) were considered significant if *p* ≤ 0.05. The *post-hoc* Bonferroni test was employed to compare differences among the treatment groups. When repeated measures ANOVA was applied, sphericity assumptions were checked by Mauchly's test and, in case of violation of sphericity, the Greenhouse-Geisser correction was used. After repeated measures ANOVA, the independent-samples *t*-test was used to compare differences between two factors or *post-hoc* two-way ANOVA was used to test for differences and interactions between two factors (WAS and DSS). Comparisons between two groups were made with the independent-samples *t*-test. In case of nonparametric distributions, the Kruskal–Wallis test was employed, and in *post-hoc* testing the Mann–Whitney *U*-test. The Bonferroni correction was used for pairwise comparisons.

## Results

### DSS-induced colitis remained unaffected by WAS

**Figure 1:**
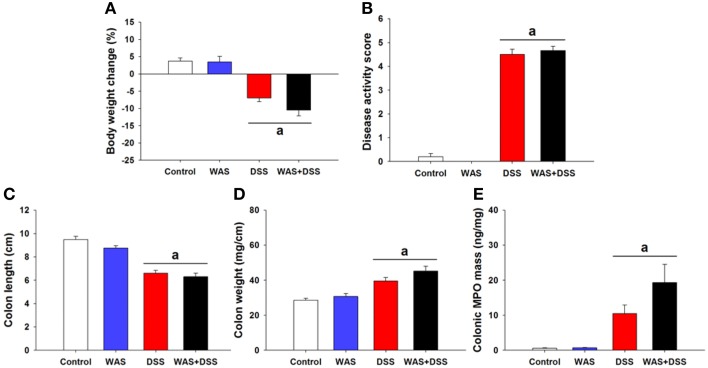
**Effect of a 7-day treatment of mice with DSS, WAS, and WAS+DSS on colitis-associated parameters measured on day 8 (*****study*** **1)**. Panel **(A)** shows percentage of body weight change relative to the weight measured before the treatment started, **(B)** disease activity score, **(C)** colon length, **(D)** colon weight, and **(E)** colonic MPO mass. Values are expressed as means + SEM or - SEM (*n* = 8–12). Two-way ANOVA did not show any WAS × DSS interaction and any WAS effect but revealed a DSS effect (^a^*p* < 0.05).

In order to study the effect of WAS on colitis induced by DSS, we examined the severity of colitis in all treatment groups (I–IV). Treatment of mice with DSS (*study* 1) induced colitis as seen in treatment groups III (DSS) and IV (WAS+DSS). Analysis of colitis-associated parameters did not reveal any significant interaction between WAS and DSS. Treatment with DSS (DSS effect *p* < 0.001) led to body weight loss (Figure 1A), increased disease activity score (Figure 1B), reduced colon length (Figure 1C) and increased colon weight (Figure 1D) as well as colonic MPO mass (Figure 1E). WAS exposure alone did not alter any of these parameters (Figures 1A–E). The changes of colitis-associated parameters shown in Figure 1 (*study* 1) were representative of those seen in the other studies (*study* 2–5). Thus, WAS did not alter colitis induced by DSS.

### WAS exposure and WAS+DSS treatment modified locomotion and rearing in a differential manner

**Figure 2:**
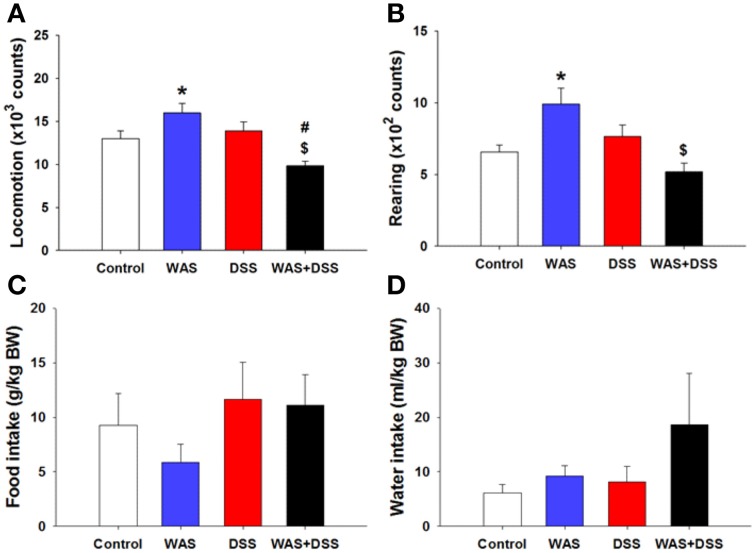
**Effect of a 7-day treatment of mice with DSS, WAS, and WAS+DSS on short-term activity (60 min) measured on day 8 (*****study*** **2)**. Panel **(A)** shows locomotion, **(B)** rearing, **(C)** food intake, and **(D)** water intake. Water and food intake are expressed relative to the body weight (BW) of the animals. Values are expressed as means + SEM (*n* = 6–8). Two-way ANOVA revealed a WAS × DSS interaction (*p* < 0.01), and *post-hoc* analysis (Bonferroni's multiple comparison) demonstrated significant differences: ^*^p < 0.05 vs. Control; ^$^*p* < 0.05 vs. WAS; ^#^*p* < 0.05 vs. DSS.

In order to examine whether DSS-induced colitis, WAS and combined WAS+DSS treatment affect spontaneous behavior in mice, we recorded short-term activity (locomotion, exploration, and ingestion) for a period of 60 min. Analysis of activity-related parameters (*study* 2) disclosed a significant interaction between WAS and DSS in their influence on locomotion and rearing (*p* < 0.01, Figures 2A,B). Bonferroni's multiple comparison revealed that WAS exposure significantly increased locomotion (*p* < 0.05 vs. control) and rearing (*p* < 0.01 vs. control), whereas DSS treatment alone had no effect. In contrast, WAS+DSS treatment led to a significant decrease in locomotion and rearing (*p* < 0.001 and *p* < 0.001 vs. WAS, *p* < 0.05 and *p* = 0.061 vs. DSS). Analysis of the food and water intake did not show any significant interaction between WAS and DSS (Figures 2C,D). Thus, WAS increased, whereas WAS+DSS treatment decreased, spontaneous activity (locomotion and rearing) in mice.

### WAS exposure improved self-care and motivational behavior

**Figure 3:**
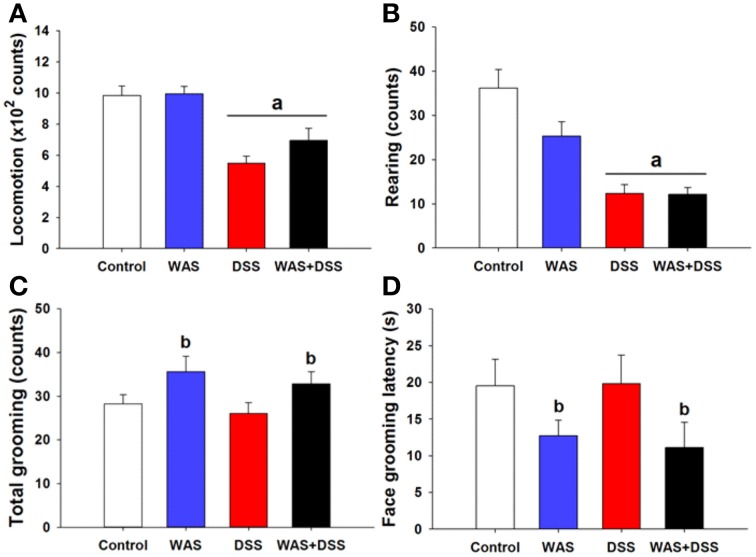
**Effect of a 7-day treatment of mice with DSS, WAS, and WAS+DSS on (A) locomotion, (B) rearing, (C) total (face + body) grooming frequency, and (D) face grooming latency recorded in the splash test performed on day 8 (*****study*** **3)**. All parameters were recorded for 5 min after spraying the mice with sucrose solution. Values are expressed as means + SEM (*n* = 8–12). Two-way ANOVA did not show any WAS × DSS interaction but revealed a DSS effect (^a^*p* < 0.05) and a WAS effect (^b^*p* < 0.05).

In order to investigate whether DSS-induced colitis, WAS and combined WAS+DSS treatment affect self-care and motivational behavior, we recorded grooming frequency and latency during the first 5 min after spraying a 10% (w/v) sucrose solution on the dorsal surface of mice. DSS treatment and WAS exposure (*study* 3) affected the animal behavior in the splash test in a disparate fashion (Figures 3A–D), although statistical analysis of the data did not show any significant interaction between WAS and DSS in their influence on locomotion, rearing, total (face + body) grooming frequency and face grooming latency. However, locomotion and rearing of DSS-treated mice in response to spraying of sucrose solution on the dorsal surface was significantly decreased (DSS effect *p* < 0.001, Figures 3A,B). The total grooming frequency was increased in WAS-exposed mice while the face grooming latency was shortened (WAS effect *p* < 0.05, Figures 3C,D). Face grooming typically preceded body grooming. Thus, WAS increased both motivational and self-care behavior of mice in the splash test.

### WAS exposure and DSS treatment increased the somatic sensitivity to mechanical and thermal stimuli

**Figure 4:**
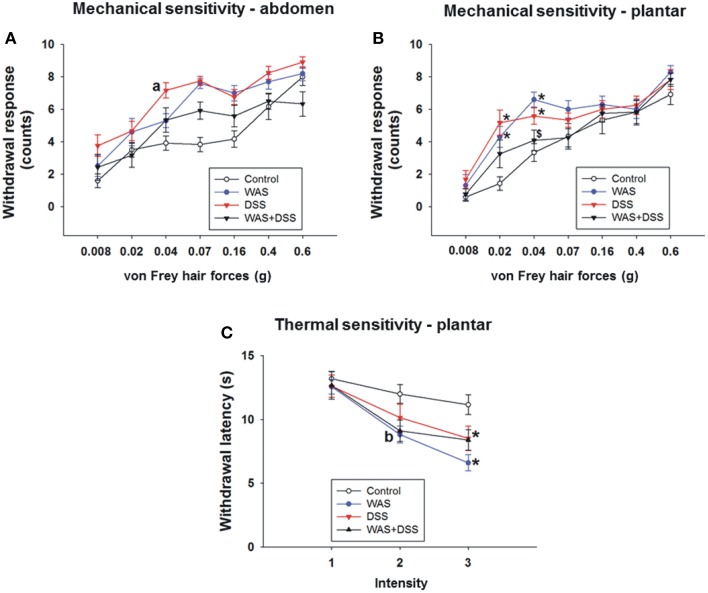
**Effect of a 7-day treatment of mice with DSS, WAS, and WAS+DSS on mechanical pain sensitivity of the abdomen (A) and plantar hind paw region (B) and on thermal pain sensitivity of the plantar hind paw region (C) recorded with the von Frey test on day 8 and the plantar test on day 9, respectively (*****study*** **4)**. Values are expressed as means ± SEM or + SEM (*n* = 10–12). Repeated measures ANOVA **(A)** did not show any WAS × DSS × forces interaction in the withdrawal response from abdominal stimulation but revealed a WAS × forces and a DSS × forces interaction (*p* < 0.05). *Post-hoc* analysis (independent-samples *t*-test) demonstrated a DSS effect (^a^*p* < 0.05) as indicated in **(A)**. Repeated measures ANOVA disclosed a WAS × DSS × forces interaction (*p* < 0.05) in the withdrawal response to mechanical plantar stimulation **(B)** and a WAS × DSS × intensity interaction (*p* < 0.05) in the withdrawal response to thermal plantar stimulation **(C)**. *Post-hoc* (two-way ANOVA) analysis demonstrated a WAS × DSS interaction at the 0.04 g force (*p* < 0.001) **(B)** and a WAS effect (^b^*p* < 0.05) at infrared intensity 1 **(C)**. *Post-hoc* (Bonferroni's multiple comparison) analysis demonstrated significant differences: ^*^p < 0.05 vs. Control; ^$^*p* < 0.05 vs. WAS.

In order to study the effect of DSS-induced colitis, WAS and combined WAS+DSS treatment on somatic nociception in mice, we assessed the sensitivity of the abdominal skin to mechanical stimuli and of the plantar skin to mechanical and thermal stimuli. Assessment of the mechanical sensitivity in the abdominal region with the von Frey test did not reveal any significant interaction between WAS/DSS and the test forces in their influence on the withdrawal response (*study* 4). There was, however, a significant interaction between WAS exposure and test forces as well as between DSS treatment and test forces in their impact on the withdrawal response (*p* < 0.05, Figure 4A). To examine these effects further, *post-hoc* analysis with the independent-samples *t*-test revealed only a DSS effect, as DSS treatment led to a significant increase in the withdrawal response to the 0.04 g force (DSS effect *p* < 0.05, Figure 4A).

Evaluation of the mechanical sensitivity of the plantar region with the von Frey test disclosed a significant interaction (*p* < 0.05, Figure 4B) between WAS/DSS and the test forces in their effect on the withdrawal response. Two-way ANOVA showed a significant interaction between WAS and DSS at three test forces; 0.02 g (*p* < 0.01), 0.04 g (*p* < 0.001, and 0.07 g (*p* < 0.05). Bonferroni's multiple comparison analysis revealed that the withdrawal response to forces of 0.02 g (*p* < 0.01 vs. control) and 0.04 g (*p* < 0.001 vs. control) was significantly increased in WAS-exposed mice. In DSS-treated animals the withdrawal response to forces of 0.02 g (*p* < 0.001 vs. control) and 0.04 g (*p* < 0.01 vs. control) was also significantly enhanced. WAS+DSS treatment led to a significant decrease in the withdrawal response to forces of 0.04 g and 0.07 g (*p* < 0.01 and *p* = 0.05 vs. WAS) and to forces of 0.02 g and 0.04 g (*p* = 0.05 and *p* = 0.054 vs. DSS) (Figure 4B).

Assessment of the thermal sensitivity on the plantar side of the hindpaw with the plantar test showed that there was a significant interaction (*p* < 0.05) between the two main factors (WAS and DSS) and the intensity of the infrared light stimulus (1, 2, and 3) in their impact on withdrawal latency (Figure 4C). While, as disclosed by *post-hoc* analysis, there was no effect of intensity 1 (data not shown) on withdrawal latency, WAS exposure caused a significant decrease in withdrawal latency (WAS effect *p* < 0.05) at intensity 2. A significant interaction (*p* < 0.05) between WAS and DSS was found at intensity 3. Bonferroni's multiple comparison revealed that the withdrawal latency was significantly decreased in both WAS-exposed and DSS-treated mice (*p* < 0.001 and *p* < 0.05 vs. control) (Figure 4C).

These data show that DSS causes mechanical hypersensitivity of the abdominal skin while both DSS and WAS induce mechanical and thermal hypersensitivity of the plantar skin. These effects were absent in animals treated with WAS+DSS.

### WAS exposure and DSS treatment induced phosphorylation of p42/44 MAPK and c-Fos expression in the spinal cord

**Figure 5:**
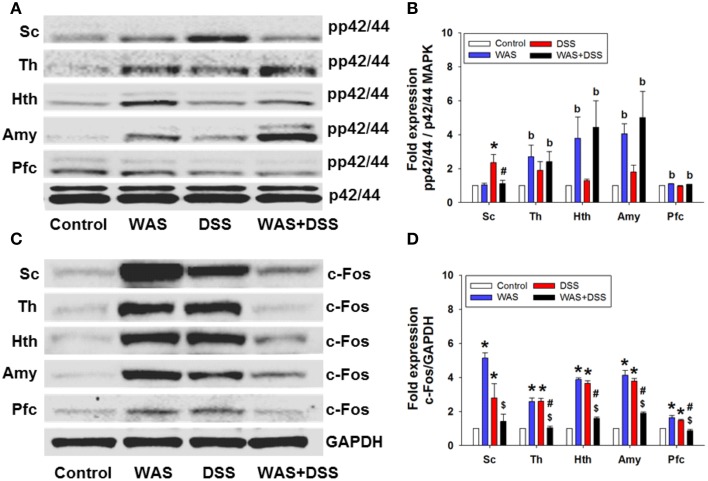
**Effect of a 7-day treatment of mice with DSS, WAS, and WAS+DSS on protein expression (A/B: phosphorylated p42/44 (pp42/44)/p42/44 MAPK, C/D: c-Fos/GAPDH) on day 8 in the spinal cord (Sc), thalamus (Th), hypothalamus (Hth), amygdala (Amy), and prefrontal cortex (Pfc) (*****study*** **1)**. Panels **(A,C)** show representative Western blots and **(B**,**D)** show quantitative data obtained by densitometric evaluation of the Western blots, expressed as x fold over control. Values shown in **(B,D)** are expressed as means + SEM (*n* = 3–4). Two-way ANOVA showed a WAS × DSS interaction (*p* < 0.05) in pp42/44 **(B)** and c-Fos **(D)** expression in the spinal cord, and a WAS × DSS interaction (*p* < 0.001) in c-Fos expression in the thalamus, hypothalamus, amygdala, and prefrontal cortex **(D)**. In addition, a WAS effect (^b^*p* < 0.05) was revealed as depicted in **(B)**. *Post-hoc* (Bonferroni's multiple comparison) analysis demonstrated significant differences: ^*^p < 0.05 vs. Control; ^$^*p* < 0.05 vs. WAS; ^#^*p* < 0.05 vs. DSS.

On the molecular level, we examined the effect of DSS-induced colitis, WAS and combined WAS+DSS treatment on p42/44 MAPK phosphorylation and c-Fos expression in the spinal cord. WAS exposure and DSS treatment (*study* 1) induced phosphorylation of p42/44 MAPK and c-Fos expression in the spinal cord (Figures 5A,C). Densitometric evaluation of the immunoreactive pp42/44 MAPK and c-Fos bands showed a significant interaction between the WAS and DSS treatments (*p* < 0.05, Figures 5B,D). Bonferroni's multiple comparison revealed that DSS treatment increased p42/44 MAPK phosphorylation (*p* < 0.01 vs. control) and c-Fos expression (*p* < 0.05 vs. control), while WAS exposure enhanced only c-Fos expression (*p* < 0.001 vs. control). WAS+DSS treatment led to a decrease in pp42/44 MAPK (*p* < 0.05 vs. DSS) and c-Fos (*p* < 0.001 vs. WAS) expression. Thus, DSS, but not WAS and WAS+DSS, caused MAPK phosphorylation in the spinal cord while c-Fos expression was stimulated by WAS and DSS, but not WAS+DSS.

### WAS exposure and DSS treatment induced phosphorylation of p42/44 MAPK and c-Fos expression in the brain

We further explored the effect of DSS-induced colitis, WAS and combined WAS+DSS treatment on p42/44 MAPK phosphorylation and c-Fos expression in the brain. WAS exposure and DSS treatment induced phosphorylation of p42/44 MAPK and c-Fos expression (*study* 1) in distinct brain areas (Figures 5A,C). Densitometric evaluation of the immunoreactive pp42/44 MAPK bands (thalamus, hypothalamus, amygdala, and prefrontal cortex) failed to show any significant interaction between the WAS and DSS treatments. WAS exposure induced p42/44 MAPK phosphorylation (Figure 5B) in the thalamus (*p* < 0.05), hypothalamus (*p* < 0.01), amygdala (*p* < 0.001), and prefrontal cortex (*p* < 0.01) (WAS effect).

WAS exposure and DSS treatment also induced c-Fos expression in specific brain areas. Densitometric evaluation of the immunoreactive c-Fos bands showed a significant interaction (*p* < 0.001) between WAS and DSS in the thalamus, hypothalamus, amygdala, and prefrontal cortex (Figure 5D). Bonferroni's multiple comparison revealed that WAS exposure and DSS treatment increased c-Fos expression in the thalamus, hypothalamus, amygdala (*p* < 0.001 vs. control) and prefrontal cortex (*p* < 0.01 vs. control). Conversely, WAS+DSS treatment decreased c-Fos expression (*p* < 0.001 vs. WAS, *p* < 0.001 vs. DSS) in the thalamus, hypothalamus, amygdala, and prefrontal cortex. Thus, WAS and WAS+DSS, but not DSS, caused MAPK phosphorylation in the brain while c-Fos expression was stimulated by WAS and DSS, but not WAS+DSS.

### Intrarectal AITC challenge induced freezing and reduced locomotion and rearing in control mice

**Figure 6:**
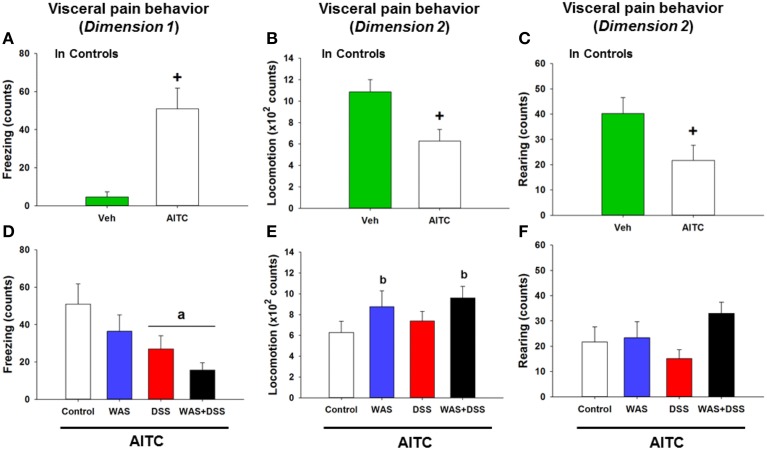
**Effect of intrarectal instillation of peanut oil (Veh) and AITC 2% (v/v) to elicit visceral pain behavior in control mice (A–C) and in mice treated with WAS, DSS, and WAS+DSS for 7 days, relative to control mice (D–F)**. The visceral pain behavior (prolonged freezing and reduced locomotion and rearing) was measured on day 8 (*study* 5). In *dimension* 1, freezing **(A,D)** was recorded for 15 min, and in *dimension* 2 locomotion **(B,E)** and rearing **(C,F)** were recorded for 5 min after intrarectal instillation. Note that the dimension of the y axis scale in **(B,E)** is ×100. Values are expressed as means + SEM (*n* = 8–12). The independent-samples *t*-test disclosed a significant difference between Veh and AITC in **(A,B)** (^+^*p* < 0.01) and **(B)** (^+^*p* < 0.05). Two-way ANOVA did not show any WAS × DSS interaction but revealed a DSS effect (^a^*p* < 0.01) and a WAS effect (^b^*p* = 0.05) with regard to changes in locomotion and rearing as indicated in **(D–F)**.

The behavioral manifestations of acute visceral pain were studied following instillation of AITC into the mouse colon. Analysis of the visceral pain behavior data (*dimension* 1 and 2, *study* 5) demonstrated that intrarectal administration of AITC to control mice prolonged their freezing behavior (*p* < 0.01 vs. vehicle, Figure 6A) while locomotion (*p* < 0.01 vs. vehicle, Figure 6B) and rearing (*p* < 0.05 vs. vehicle, Figure 6C) were significantly shortened.

### Intrarectal AITC challenge reduced freezing in DSS-treated mice and induced locomotion and rearing in WAS-exposed mice

In order to examine whether DSS-induced colitis, WAS and combined WAS+DSS treatment affect visceral nociception in mice, pain-related behavior was recorded during the first 5 min after intrarectal AITC administration. The visceral pain behavior evoked by AITC (*study* 5) was modified by WAS exposure and DSS treatment in a differential manner, although there was no significant interaction between WAS and DSS in their influence on AITC-evoked changes of freezing, locomotion, and rearing in the treatment groups. The freezing counts were significantly decreased in DSS-treated mice (DSS effect *p* < 0.01, Figure 6D) while locomotion (WAS effect *p* = 0.05, Figure 6E) and rearing (WAS effect *p* = 0.06, Figure 6F) were prolonged in WAS-exposed mice. Thus, DSS reduced freezing and WAS increased locomotion in response to intrarectal AITC administration in mice.

### WAS exposure and DSS treatment modified AITC-evoked phosphorylation of p42/44 MAPK and c-Fos expression in the spinal cord and brain

**Figure 7:**
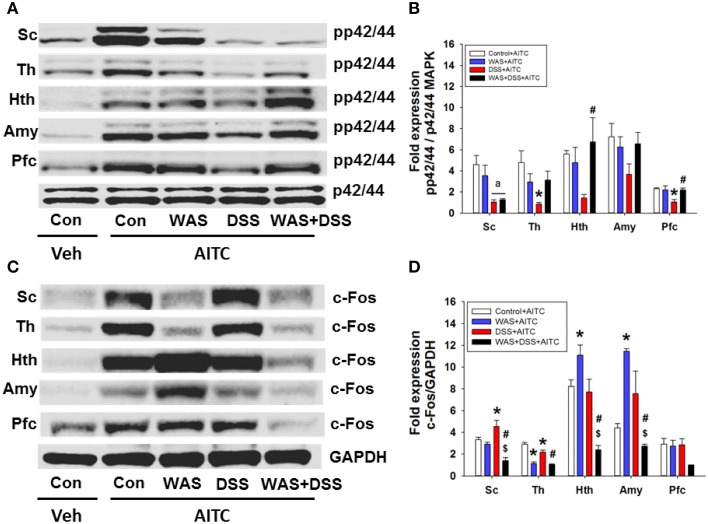
**Effect of intrarectal instillation of peanut oil (Veh) and AITC 2% (v/v) on protein expression (A/B: phosphorylated p42/44 (pp42/44)/p42/44 MAPK, C/D: c-Fos/GAPDH) in the spinal cord (Sc), thalamus (Th), hypothalamus (Hth), amygdala (Amy), and prefrontal cortex (Pfc) of control (Con) mice and mice treated with WAS, DSS, and WAS+DSS for 7 days (*****study*** **5)**. Protein expression was evaluated on day 8. Panels **(A,C)** show representative Western blots and **(B,D)** show quantitative data obtained by densitometric evaluation of the Western blots, expressed as x fold over control. Values shown in **(B,D)** are expressed as means + SEM (*n* = 3–4). Two-way ANOVA demonstrated a WAS × DSS interaction in pp42/44 expression **(B)** in the thalamus (*p* < 0.05), hypothalamus (*p* < 0.05), and prefrontal cortex (*p* < 0.05) and in c-Fos expression **(D)** in the spinal cord (*p* < 0.01), thalamus (*p* < 0.05), hypothalamus (*p* < 0.001), and amygdala (*p* < 0.05). In addition, a DSS effect (^a^*p* < 0.05) was revealed as depicted in **(B)**. *Post-hoc* (Bonferroni's multiple comparison) analysis demonstrated significant differences: ^*^p < 0.05 vs. Control+AITC; ^$^*p* < 0.05 vs. WAS+AITC; ^#^*p* < 0.05 vs. DSS+AITC.

In a subsequent experiment we investigated the effect of DSS-induced colitis, WAS and combined WAS+DSS treatment on the AITC-induced p42/44 MAPK phosphorylation and c-Fos expression in the spinal cord and brain. The effect of intrarectal AITC challenge (*study* 5) to induce phosphorylation of p42/44 MAPK and expression of c-Fos in the spinal cord and brain, as seen in control mice, was altered by DSS, WAS, and WAS+DSS treatment in a region-related manner (Figures 7A,C). Densitometric evaluation of the immunoreactive bands revealed a significant interaction between WAS and DSS with respect to AITC-evoked levels of pp42/44 MAPK in the thalamus (*p* < 0.05), hypothalamus (*p* < 0.01), and prefrontal cortex (*p* < 0.05) (Figure 7B). Bonferroni's multiple comparison revealed that the effect of AITC to enhance p42/44 MAPK phosphorylation in the thalamus (*p* < 0.01), hypothalamus (*p* = 0.05), and prefrontal cortex (*p* < 0.01) was mitigated in DSS-treated mice (vs. control mice). This inhibitory effect of DSS treatment on the AITC response in the brain was reversed by concomitant WAS exposure (WAS+DSS). When compared to DSS-treated mice, phosphorylation of p42/44 MAPK was increased in the thalamus (*p* = 0.06), hypothalamus (*p* < 0.05), and prefrontal cortex (*p* < 0.01) of WAS+DSS-treated mice (Figure 7B). There was no significant interaction between WAS and DSS with regard to the AITC-evoked pp42/44 MAPK expression in the spinal cord. However, DSS treatment decreased AITC-evoked pp42/44 MAPK levels in the spinal cord (*p* < 0.05) (DSS effect). Thus, DSS reduced AITC-evoked MAPK phosphorylation in the thalamus, hypothalamus, and prefrontal cortex, an effect that was absent in animals treated with WAS+DSS.

Densitometric evaluation of the immunoreactive c-Fos bands (Figure 7C) revealed a significant interaction between WAS and DSS with regard to the AITC-evoked c-Fos expression in the spinal cord (*p* < 0.01), thalamus (*p* < 0.05), hypothalamus (*p* < 0.001), and amygdala (*p* < 0.05) (Figure 7D). Bonferroni's multiple comparison showed that the effect of AITC to stimulate c-Fos expression was decreased in the thalamus (*p* < 0.001), but increased in the hypothalamus (*p* < 0.05) and amygdala (*p* < 0.001) of WAS-exposed animals (vs. control animals). The effect of AITC to induce c-Fos expression in the thalamus was diminished (*p* < 0.01) in DSS-treated animals, but enhanced (*p* < 0.05) in the spinal cord (vs. control mice, Figure 7D). The effects of WAS exposure and DSS treatment on the c-Fos response to AITC were reversed in the spinal cord and particular brain regions when the two treatments were combined (Figure 7D). Thus, in mice treated with WAS+DSS, the AITC-evoked expression of c-Fos in the spinal cord, thalamus, hypothalamus (*p* < 0.001) and amygdala (*p* < 0.01) was decreased relative to animals treated with DSS. Moreover, c-Fos expression was diminished in the spinal cord (*p* < 0.01), hypothalamus (*p* < 0.01) and amygdala (*p* < 0.001) of mice treated with WAS+DSS (vs. WAS). Taken together, DSS, WAS and WAS+DSS treatment altered AITC-evoked expression of c-Fos in the spinal cord and brain in a treatment- and region-dependent manner.

### Morphine modified the effects of AITC on freezing, locomotion, and rearing in control mice

**Figure 8:**
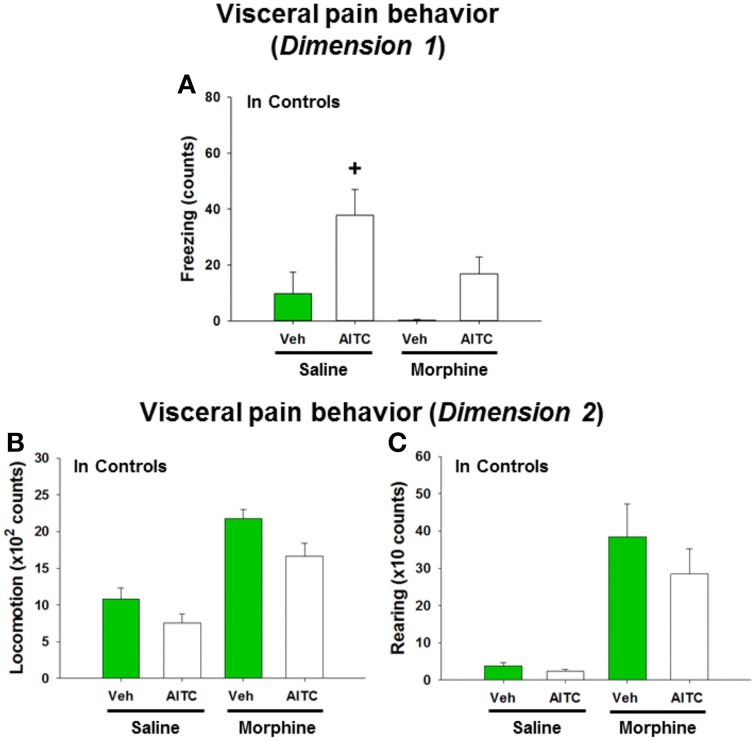
**Visceral pain behavior (freezing, locomotion and rearing) in control mice (A–C) following intrarectal instillation of peanut oil (Veh) or AITC 2% (v/v) in the absence or presence of morphine**. Saline or morphine (10 mg/kg, s.c.) was injected 1 h prior to intrarectal instillation. The visceral pain behavior was measured on day 8 (*study* 6). In *dimension* 1, freezing **(A)** was recorded for 15 min, and in *dimension* 2 locomotion **(B)** and rearing **(C)** were recorded for 5 min after intrarectal instillation. Please note that the dimension of the y axis scale **(B,C)** is ×100 and ×10, respectively. Values are expressed as means + SEM (*n* = 7–8). Data in **(A)** were evaluated with the Kruskal–Wallis one-way ANOVA test (*p* < 0.001), and the Mann–Whitney *U*-test (*post-hoc* test) demonstrated a significant difference between Veh and AITC in saline-injected control mice (^+^*p* < 0.05).

In order to address the question whether the behavioral and molecular CNS responses to intrarectal AITC administration are related to pain, control mice were injected with morphine prior to AITC instillation. Morphine had distinct effects on the behavior of mice (*study* 6). The freezing counts recorded in morphine-treated control mice were not normally distributed, and analysis of the data with a non-parametric test revealed significant differences in freezing behavior (*p* < 0.001, Figure 8A) among the treatment combinations (saline + peanut oil, saline + AITC, morphine + peanut oil, morphine + AITC) in control animals. *Post-hoc* analysis with the Mann–Whitney *U*-test confirmed that AITC instillation in saline-injected control mice prolonged freezing (*p* < 0.05 vs. saline + peanut oil). Morphine caused an approximately 50% (although non-significant) reduction of freezing counts in AITC-treated mice (Figure 8A).

Locomotion and rearing were likewise affected by morphine. In morphine-injected control mice a 2-fold increase in locomotion and approximately 10-fold increase in rearing was observed when compared to saline-injected control mice (Figures 8B,C). The extensive increase in rearing was most likely a recording artifact caused by the morphine-evoked Straub phenomenon (upright position of the tail) (Faenzi, 1961). The behavioral parameters recorded after peanut oil instillation to saline-injected mice did not significantly differ from those recorded without prior saline injection (compare Figure 8 with Figures 6A–C). Taken all findings together, the effect of morphine on the AITC-evoked pain behavior proved difficult to evaluate because morphine *per se* increased the activity of the mice.

### Morphine blunted the AITC-evoked phosphorylation of p42/44 MAPK and c-Fos expression in the spinal cord and brain of control mice

**Figure 9:**
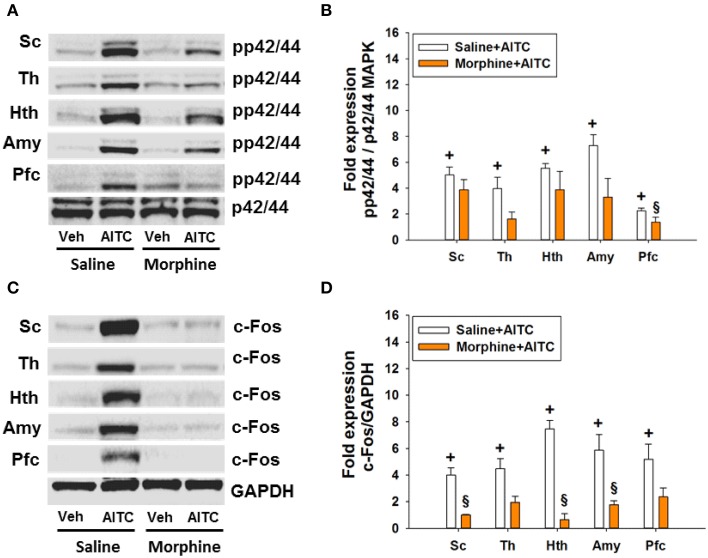
**Protein expression (A/B: phosphorylated p42/44 (pp42/44)/p42/44 MAPK, C/D: c-Fos/GAPDH) in the spinal cord (Sc), thalamus (Th), hypothalamus (Hth), amygdala (Amy), and prefrontal cortex (Pfc) of control mice following intrarectal instillation of peanut oil (Veh) or AITC 2% (v/v) in the absence or presence of morphine (*****study*** **6)**. Saline or morphine (10 mg/kg, s.c.) was injected 1 h prior to intrarectal instillation. Panels **(A,C)** show representative Western blots, and **(B,D)** show quantitative data obtained by densitometric evaluation of the Western blots, expressed as x fold over control. Values shown in **(B,D)** are expressed as means + SEM (*n* = 3–4). The graph compares the AITC-induced protein expression levels in control animals (Veh + Con in Figure 7 pooled with Saline + Veh in Figure 9) with those in morphine-treated animals (Morphine + Veh in Figure 9). Values are given as means + SEM (*n* = 3–6). Analysis with the independent-samples *t*-test disclosed significant differences: ^+^*p* < 0.05 vs. Saline+Veh; ^§^*p* < 0.05 vs. Saline+AITC.

The AITC-induced levels of both MAPK and c-Fos were markedly diminished in morphine-injected control animals (Figures 9A,C). Densitometric evaluation of the immunoreactive bands confirmed the effect of AITC to increase (*p* < 0.05 vs. vehicle) the phosphorylation of p42/44 MAPK and the expression of c-Fos (Figures 9B,D) in the spinal cord and all brain regions examined. Morphine blunted the AITC-evoked rise of c-Fos and pp42/44 MAPK levels in the brain and spinal cord in a region-dependent manner (Figures 9B,D). The expression of c-Fos and pp42/44 MAPK in the CNS measured after peanut oil instillation to saline-injected mice did not significantly differ from that measured without prior saline injection (compare Figure 9 with Figure 7). Taken all data together, morphine reduced the effect of intrarectal AITC to evoke freezing, MAPK phosphorylation and c-Fos expression in the CNS of control mice.

## Discussion

Chronic visceral pain in inflammatory bowel disease and irritable bowel syndrome can be exaggerated by stress, but the mechanisms behind this relationship are little understood. We therefore examined in which way DSS-induced colitis and repeated WAS, a psychological stressor, interact in shaping visceral pain-related behavior and molecular signaling in the CNS. In the interpretation of WAS/DSS interactions it need be emphasized that WAS failed to alter the severity of DSS-induced colitis as judged by body weight, colon length, colon weight, colonic MPO content, and disease activity score (Figures 1A–E). Although repeated WAS can disturb gastrointestinal function (Soderholm et al., 2002; Velin et al., 2004), our findings confirm other reports that WAS does not modify experimental colitis in rodents (Deiteren et al., 2014; Hassan et al., 2014). We hence conclude that WAS/DSS interactions with respect to pain-related behavior and molecular signaling in the CNS do not reflect WAS-induced alterations of colitis but take place within the brain.

### Colitis and WAS result in alterations of spontaneous behavior and CNS signaling

Inflammation and psychological stress modify behavior related to emotionality, pain (Cattaruzza et al., 2013; Banozic et al., 2014) and sickness (Zhang et al., 2014; Farzi et al., 2015). Increased activity in rats exposed to unpredictable chronic mild stress resembles the psychomotor agitation seen in patients with psychiatric disorders (Gronli et al., 2005). Here we found that WAS increased, whereas WAS+DSS treatment decreased, spontaneous activity (locomotion and rearing) in mice (Figures 2A,B). It should be noted that these alterations of spontaneous behavior included a component of novelty-induced behavior since the animals were not habituated to the test cages. The behavioral perturbations occurred independently of changes in ingestion (Figures 2C,D) and thus do not reflect sickness but are rather of an emotional-affective nature. Repeated WAS has been argued to represent a model of predictable chronic stress (Hassan et al., 2014), which may explain why WAS increased both motivational and self-care behavior in the splash test (Figures 3A–D). This beneficial influence of WAS is consistent with its ability to counteract the effects of DSS-induced colitis to reduce social interaction and elevate anxiety in association with increased plasma corticosterone and hypothalamic neuropeptide Y mRNA expression (Hassan et al., 2014).

WAS and DSS were also found to alter c-Fos expression and MAPK phosphorylation in the CNS independently of any acute intervention (Figures 5A–D). These changes in CNS signaling are most likely related to the alterations of spontaneous behavior in WAS-exposed and DSS-treated mice. The induction of c-Fos occurs downstream of MAPK stimulation but is also regulated by other signaling pathways (Kovacs, 1998; O'Donnell et al., 2012; Reul, 2014), which is consistent with the dissimilar MAPK and c-Fos activation patterns in the different treatment groups. While both DSS and WAS induced c-Fos in the spinal cord, thalamus, hypothalamus, amygdala, and prefrontal cortex, cerebral MAPK phosphorylation was not affected by DSS but enhanced by WAS. We surmise that p42/44 MAPK activation is of particular relevance to the emotional and motivational alterations seen in WAS-exposed mice.

It is worth noting that combined WAS+DSS treatment induced less c-Fos in the CNS than WAS or DSS separately (Figures 5C,D). This observation may be related to the finding that WAS+DSS treatment increases circulating corticosterone to a larger extent than WAS or DSS alone (Hassan et al., 2014), given that dexamethasone inhibits c-Fos induction by stress and inflammatory pain (Buritova et al., 1996; de Medeiros et al., 2005). Such a mechanism may also apply to the effect of WAS+DSS to decrease locomotion whereas WAS enhances this parameter and DSS has no effect (Figure 2A). Since, in addition, WAS prevents the anxiogenic effect of DSS (Hassan et al., 2014), we assume that WAS-evoked locomotion reflects psychomotor agitation which is mirrored by MAPK activation and c-Fos expression in the CNS.

### Central sensitization evoked by WAS and colitis is mirrored by alterations in CNS signaling

Visceral hypersensitivity to colonic distention develops after DSS or WAS exposure (Larauche et al., 2011; Chen et al., 2013; Deiteren et al., 2014), and the present findings show that DSS causes mechanical hypersensitivity of the abdominal skin while both DSS and WAS induce mechanical and thermal hypersensitivity of the plantar skin. This observation is indicative of somatic hyperalgesia, whereas the DSS-induced mechanical hyperalgesia of the abdominal skin reflects most likely referred pain associated with experimental colitis (Laird et al., 2001; Eijkelkamp et al., 2007) because WAS failed to alter the mechanical pain sensitivity of the abdomen. The presence of somatic hyperalgesia in mice with DSS-induced colitis is consistent with the presence of somatic hyperalgesia in irritable bowel syndrome patients (Stabell et al., 2013). Since both colitis and WAS induce visceral and/or somatic pain hypersensitivity, we conclude that pain sensitization takes place at the CNS level.

The literature regarding the effect of WAS on visceral pain is inconsistent. Several reports attest to WAS-induced mechanical hypersensitivity in male Wistar, Long-Evans and Fischer-344 rats (Bradesi et al., 2005; Schwetz et al., 2005; Johnson et al., 2015), although other reports hold that repeated exposure to WAS does not alter mechanical sensitivity in female Wistar rats (Deiteren et al., 2014) or even causes immediate post-stress hypoalgesia in female Wistar rats (Larauche et al., 2012) and male Long-Evans rats (Schwetz et al., 2005). Our and another study in male and female mice (Larsson et al., 2009), respectively, failed to observe any repeated WAS-induced mechanical hypersensitivity, which could relate to the absence of changes in corticotropin-releasing factor (CRF) and glucocorticoid receptor mRNA expression in the mouse amygdala (Hassan et al., 2014). In male Fischer-344 rats, however, both CRF and glucocorticoid signaling in this region contribute to visceral hypersensitivity (Myers and Greenwood-Van Meerveld, 2012; Johnson et al., 2015). Species, strain and sex differences need therefore to be considered as factors that influence the ability of WAS to affect visceral sensitivity. In female Wistar rats, the failure of repeated WAS to cause visceral hyperalgesia is associated with a failure to activate the hypothalamic-pituitary-adrenal axis (Deiteren et al., 2014). In male Long-Evans rats, acute exposure to WAS induces visceral hypersensitivity only in maternally separated animals, an effect that seems to involve central, but not peripheral, CRF receptors (Schwetz et al., 2005; van den Wijngaard et al., 2012). The potential influence of sex was examined in Wistar rats in which Larauche et al. (2012) observed visceral analgesia immediately after acute WAS exposure, an effect that depended on opioid signaling in female, but not male, animals. Apart from species, strain and sex, factors such as the method used to record visceral sensitivity, housing condition and animal handling may in addition affect WAS-associated changes in visceral sensitivity.

The mechanical hyperalgesia in the abdominal skin of DSS-treated mice and the mechanical and thermal hyperalgesia in the plantar skin of DSS- and WAS-treated mice failed to develop when the animals were treated with WAS+DSS. This observation suggests that DSS- and WAS-induced sensitization processes involve different signaling mechanisms that interact with each other in a negative way. In keeping with this contention, WAS and WAS+DSS, but not DSS, caused MAPK phosphorylation in the brain while c-Fos expression was stimulated by WAS and DSS, but not WAS+DSS. These results suggest that DSS and WAS activate different signal transduction pathways that converge on c-Fos but inhibit each other in inducing c-Fos when activated in parallel.

The ability of WAS and DSS to cause MAPK and/or c-Fos activation is most probably of relevance to central pain sensitization. This conclusion is backed by the findings that the mechanical and thermal hyperalgesia in the abdominal or plantar skin of DSS- or WAS-treated mice as well as the DSS- and WAS-induced c-Fos expression in the CNS disappeared when the animals were treated with WAS+DSS. Importantly, MAPK phosphorylation and c-Fos induction were altered in the absence of any acute intervention and thus may have a bearing on WAS- and DSS-evoked alterations of spontaneous behavior.

### Colitis and WAS alter cerebral processing of acute visceral pain in a differential manner

Apart from alterations of spontaneous behavior and CNS signaling, WAS and DSS modified the cerebral responses to an acute visceral pain stimulus, intrarectal AITC. This chemical stimulus is known to elicit pain and activate p42/44 MAPK and c-Fos in the lumbosacral spinal cord within 1 h after administration (Galan et al., 2003; Mitrovic et al., 2010). Here we found that intrarectal AITC also activated p42/44 MAPK and c-Fos in all brain regions examined. These signaling reactions were associated with prolonged freezing (Laird et al., 2001) and decreased activity, thought to be a readout of visceral pain in mice (Schwartz et al., 2013). We hypothesize that MAPK and c-Fos activation in the CNS mirrors the sensory, emotional and autonomic processing of a visceral pain stimulus. This contention is affirmed by the ability of morphine to inhibit both AITC-evoked aversive behavior and molecular signaling in the CNS.

Experiments involving DSS and WAS enabled us to compare the cerebral processing of an acute visceral pain stimulus under conditions of hyperalgesia with and without colonic inflammation. While DSS and WAS modified molecular brain signaling, DSS reduced pain-related freezing (a DSS effect). WAS and DSS likewise failed to modify the visceromotor pain response to colorectal distention in mice (Larsson et al., 2009). In contrast, AITC-associated locomotion and c-Fos expression in the hypothalamus and amygdala were enhanced by WAS whereas c-Fos expression in the thalamus was diminished. A similar region-specific effect emerges from functional brain mapping studies in rats exposed to WAS (Wang et al., 2013) and in irritable bowel syndrome patients (Tillisch et al., 2011), which points to exaggerated emotional regulation of distention-evoked colorectal pain. Unlike WAS, DSS blunted the AITC-evoked phosphorylation of p42/44 MAPK in the absence of any major effect on c-Fos expression and inhibited the freezing reaction to AITC. We conclude that DSS-induced colitis leads to inhibition of cerebral MAPK signaling which is unrelated to c-Fos expression but linked to pain-related behaviors such as freezing. This contention conforms with the ability of MAPK inhibitors to prevent visceral pain behavior (Matsuoka and Yang, 2012; Galan-Arriero et al., 2014) and re-emphasizes that MAPK activation rather than c-Fos expression is relevant to nociception-related behavior.

A differential engagement of MAPK and c-Fos signaling in visceral pain behavior is further corroborated by the effect of combined WAS+DSS treatment which blunts the freezing reaction to intrarectal AITC (a DSS effect), prolongs the locomotor reaction (a WAS effect) and causes distinct changes in molecular CNS signaling. While the inhibitory effect of DSS on cerebral MAPK phosphorylation was neutralized by WAS, combined WAS+DSS treatment inhibited AITC-evoked c-Fos expression in all CNS regions examined. These observations indicate that experimental colitis and repeated WAS impact on different signaling pathways in brain areas relevant to visceral pain processing. They also show that stress can counteract the molecular manifestations of DSS-induced colitis in the brain, which could explain why repeated WAS prevents the effect of DSS to enhance anxiety and reduce social interaction (Hassan et al., 2014).

### The effect of morphine indicates that AITC-evoked changes in p42/44 MAPK and c-Fos activation are related to nociception

The ability of morphine to reduce the effect of intrarectal AITC to evoke freezing, MAPK phosphorylation and c-Fos expression in the CNS in control mice indicates that these responses involve opioid-sensitive neural pathways and reflect manifestations of visceral nociception. The effect of AITC to inhibit MAPK phosphorylation in the hypothalamus and amygdala was relatively resistant to inhibition by morphine. It is unlikely that this finding is related to the action of morphine to stimulate locomotion and induce the Straub phenomenon because morphine blunted the AITC-evoked c-Fos expression in all CNS areas of all treatment groups. These observations reaffirm the contention that MAPK and c-Fos signaling make differential contributions to visceral pain processing in the CNS. As previously proposed (Mitrovic et al., 2010), the site of action of morphine is thought to be within the CNS because the colitis parameters did not differ between the different treatment groups.

## Conclusions

This study provides new perspectives on the cerebral processing of visceral pain and the differential impact of colitis and psychological stress on pain-related behavior and cerebral p42/44 MAPK and c-Fos activation. DSS and WAS act on different signaling pathways that converge on c-Fos in a stimulatory or inhibitory fashion, modifying spontaneous behavior and the behavioral and molecular responses to an acute visceral pain stimulus in a differential manner. The involvement of p42/44 MAPK and c-Fos in different aspects of visceral pain emphasizes the need for investigating the molecular and behavioral manifestations of pain in parallel in order to understand the sensory and emotional processing of a visceral pain stimulus. Further work is required to define the precise relationship between cerebral MAPK and c-Fos signaling and distinct behavioral manifestations of visceral pain.

## Conflict of interest statement

The authors declare that the research was conducted in the absence of any commercial or financial relationships that could be construed as a potential conflict of interest.
